# “I-We-I”: Visualizing Adolescents’ Perceptions and Apprehension to Transition to Adult HIV Care at a Supportive Transition Facility in the Cape Town Metropole, South Africa

**DOI:** 10.3390/tropicalmed10050126

**Published:** 2025-05-06

**Authors:** Charné Petinger, Brian van Wyk, Talitha Crowley

**Affiliations:** 1 School of Public Health, University of the Western Cape, Cape Town 7535, South Africa; bvanwyk@uwc.ac.za; 2 School of Nursing, University of the Western Cape, Cape Town 7535, South Africa; tcrowley@uwc.ac.za

**Keywords:** ALHIV, HIV, transitions of care, photovoice, South Africa

## Abstract

Adolescents living with HIV (ALHIV) (10–19 years) make up approximately 4.2% (320,000) of people living with HIV in South Africa. Adolescence is a developmental period characterized by pervasive biological, social and psychological changes, which challenges adherence and retention in care for ALHIV on antiretroviral therapy (ART). Further, as ALHIV grow “older”, they are expected to transition to the adult HIV treatment programme, where they should assume greater responsibility for managing their chronic condition and healthcare pathway. Whereas it is imperative that ALHIV are transitioned when they are ready, little is known about the challenges and experiences of ALHIV before and during transition. The aim of this paper was to report on the experiences and challenges of transition for ALHIV who received ART at an adolescent-friendly service that is adjunct to a public primary healthcare facility in the Western Cape province of South Africa. Methods: Photovoice methods were employed to explore the transition experiences of ALHIV on ART at a “supportive transition” public health facility in the Cape Town Metro in South Africa. Participants took pictures that depict their experience pre- and during transition to adult care and discussed these in groups with peers. Audio data were digitally recorded and transcribed verbatim and subjected to thematic analysis using Atlas.Ti version 24. Results: The emergent themes described their apprehension to transitioning to adult care; self-management; challenges to adherence; the need for psychosocial support; and how adolescent-friendly services were filling the gap. Conclusions: We illuminate the “I-We-I” configuration, to reflect (the first “I”) individual ALHIV experiences as isolated before being transferred to the supportive facility; how they experience a sense of belonging and family (“we”) in the supportive facility; but face apprehension about transitioning to adult care in the local clinic, where they have to self-manage (final “I”).

## 1. Introduction

Globally, there are estimated 39.9 million people living with HIV (PLHIV) in 2023, whereof adolescents living with HIV (aged 10–19 years) account for 1.7 million. Notably, 82% of these adolescents reside in Sub-Saharan Africa [1,2]. In South Africa, ALHIV make up an approximate 4.2% (320,000) of the HIV population [3,4].

Adolescence is a developmental period characterized by pervasive biological, social and psychological changes [5]. As a result of the successful rollout of antiretroviral therapy (ART), ALHIV are surviving and are expected to live longer, healthier lives. Subsequently, this requires lifelong treatment as they are maturing into adulthood. However, in the face of the unique challenges of adolescence, it is widely reported that ALHIV exhibit decreased adherence to treatment and lower retention in care [6,7] compared to children and adults [8,9,10,11], which result in higher rates of HIV-related comorbidities and mortality [12].

It is estimated that approximately 320,000 ALHIV will transition from paediatric to adult ART care by 2028 in South Africa [3,4]. The transition to adult HIV care is considered to be a vulnerable period, which coincides with lower adherence and viral suppression rates [13]. The transition process can be described as a shift from caregiver-led treatment (in paediatrics) to autonomous treatment-taking and care (in adult care) [14]. Additionally, the healthcare transition requires a coordinated negotiation between the patient, their parents or caregivers, the paediatric healthcare provider and the adult healthcare provider to ensure that autonomous health-related decision-making is achieved [15]. The transition varies significantly, as paediatric settings offer ALHIV individualised care and closer relationships with healthcare providers, whereas adult care is often characterised by greater independence [14,16]. Consequently, ALHIV must navigate the shift from a familiar, supportive system to a potentially unfamiliar one and demonstrate independence with respect to their care pathway within an overburdened health system [12,17].

Transitioning to adult care involves taking charge of their own health and disease management, which raises the personal challenge of persistent engagement in care for ALHIV, as they might get “lost” in the health system [18]. It is therefore imperative that ALHIV are transitioned when they are ready, being adequately prepared and guided in the process of assuming self-responsibility for their care [19,20]. A recent review reports that there are few transition policies and guidelines in place at both national and operational levels [21]. Whilst there are available guidelines on transferring paediatric patients to an adult HIV clinical regimen, there is a lack of sufficient guidance on the shift of responsibility encompassing the transition to adult HIV services [22,23,24]. In South Africa, with a high burden of HIV amongst adolescents, the challenge to transition successfully is exacerbated by overburdened and poorly functioning health systems and “deficient” models of care for ALHIV [25]. This warrants the need for exploring the transition experiences of ALHIV at public health care facilities, to effectively identify the strengths and gaps in the provision of care. The aim of this paper was to report on the experiences and challenges of transition for ALHIV who received ART at an adolescent-friendly service that is adjunct to a public primary healthcare facility in the Western Cape province of South Africa.

## 2. Materials and Methods

### 2.1. Study Design

This study utilized photovoice methodology to explore the transition experiences of ALHIV on ART. Photovoice is a participatory research method, which allows participants to depict and express their experiences and feelings through pictures. Photovoice can therefore be beneficial for ALHIV through providing an opportunity to share complex emotions and experiences. Through the integration of pictures and narratives, photovoice assisted in this research to explore the participants’ meaning-making of the transition process and their experiences at different facilities [26].

### 2.2. Study Context

**Figure 1:**
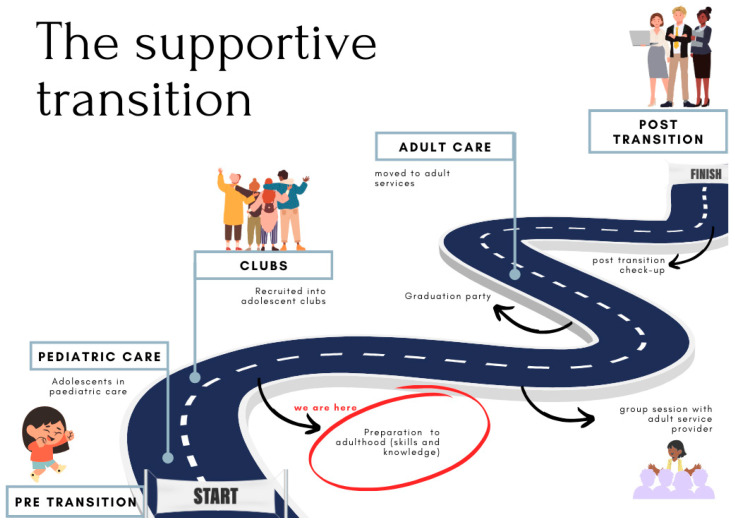
Supportive transition facility [25]. The red circle indicates where in the transition process ALHIV are currently at.

This study was conducted in the Cape Town Metropole, which houses an approximate 637,353 adolescents between the ages of 10 and 19 years. In 2022, the Metropole had a population of 540,000 PLWHIV, of which 4.3% were ALHIV [27]. The facility where this study was conducted offers services in an urban, high-density area to a population of 200,000 people [6]. This facility (Figure 1) can be described as a *supportive transition facility* [25]. The facility provides dedicated care to ALHIV, aged 10–24 years old, in the form of youth clubs wherein peer support, mentoring and close relationships with the healthcare providers are prioritized. ALHIV were referred to the facility by their paediatric physician at the local public primary healthcare clinic after being identified as sufficiently adherent, an adolescent and assessed as likely to benefit from the social aspects of care being provided at the *supportive transition* facility [25]. The services that they are provided with include after school clinic visits, group sessions focused on sharing experiences, knowledge and life skills preparation, WhatsApp groups and access to healthcare workers outside of their scheduled clinic visits and camps to foster peer relationship-building and skills building.

### 2.3. Sampling and Participants

Participants were ALHIV who were on ART and receiving care in this adolescent-specific supportive facility. Participants were therefore purposefully sampled, after identification by key staff members in the facility took place. During their scheduled group dates, the research team presented the study to the potential participants. The inclusion criteria for eligible participants were that they were living with HIV, disclosed to, receiving care at the selected facility, between the ages of 10 and 19 years at the time of the study and willing to take part in the study and follow-up sessions in a group setting, as set out by information sheets and consent forms. Recruitment took place over the course of three weeks. Twenty-four participants completed all three photovoice discussions, wherein 16 (66.7%) were between the ages of 16 and 19 years, and 15 participants (62.5%) were female. See Table 1 below for a description of the study participants:

### 2.4. Procedures

The data collection team, comprising of the first author and research assistant, received photovoice training from an experienced HIV researcher with photovoice experience. Each photovoice project consisted of three sessions. In the first session, recruitment of participants took place. During this session, researchers provided eligible participants information sheets and consent forms in the language of their choice and explained the purpose of the study. In the next session, the participants were required to bring back signed consent forms (and parental consent forms if under the age of 18 years). After providing the signed forms, the first author provided the cell phones with cameras to participants and explained the study thoroughly. Additionally, we provided an instruction sheet explaining that participants cannot take pictures of themselves or other people (in terms of POPI Act 4 of 2013) and advised them to prioritise their safety and that of their phones, given that many participants come from marginalised communities. Participants were then told to take 3–5 pictures of anything that represents the care that they are currently receiving, how they feel about growing up and going to an adult facility as ALHIV (including any self-management strategies). At the third and final session, participants had to select three pictures to discuss in a group setting and provide a caption for each picture as a group.

### 2.5. Data Collection

Data collection was conducted between September and November 2024. Participants took pictures of their experiences explained and discussed them as a group. In these focus group sessions, the pictures were displayed through a mobile projector operated by the researchers. The focus groups were centred around participants explaining their pictures with the first author facilitating the discussion to highlight affirming or opposing views. Each focus group consisted of 2–6 participants of all genders and lasted between 30 and 120 min. The sessions were recorded and transcribed verbatim by the first author (CP).

### 2.6. Data Analysis

The transcripts of the recorded focus groups were uploaded to Atlas.Ti version 24 and subjected to reflexive thematic analysis by the first author (CP). The steps of reflexive thematic analysis as set out by Braun and Clarke were followed [28,29]. Reflexive thematic analysis was beneficial to this photovoice study as it enables a flexible and in-depth exploration into the transition experiences of ALHIV while prioritizing their lived realities. Prior to analysis, the pictures were uploaded to the transcripts at the timestamps they were discussed.

### 2.7. Rigour

Trustworthiness for this study was maintained through the concepts set forth by Lincoln and Guba [30]. This included credibility, which is being maintained through thick, rich descriptions of the data [31]. Secondly, dependability was achieved through following the abovementioned procedures for the data collection [30]. Confirmability was maintained by the first author (CP) keeping a reflective journal throughout the process, as well as peer debriefing with the research team. The journaling and discussions facilitated critical examinations of our assumptions and potential biases. Moreover, participants’ voices were centred through the use of direct quotes, their pictures and captions to support our findings and enhance confirmability. Lastly, transferability was maintained through rich descriptions of the study context and the participants, which can be used in future research [30].

### 2.8. Ethics Considerations

Ethics clearance was obtained at the first author’s (CP) registered university, as this study formed part of the first author’s doctoral research project (BM23/6/5). Permission to access the selected facility was granted by the Provincial Health Research Committee (WC_202308_043). Participants were provided with information sheets and consent and assent forms (for participants under the age of 18 years) prior to data collection. This ensured that participants were aware of their rights as research participants, such as being fully informed what their participation would entail, that they are free to withdraw from the study without any negative consequences and that their personal information will be kept anonymous and confidential. The use of pseudonyms throughout the entire research ensured that confidentiality and anonymity were maintained. The collected data were stored in a secure, password-protected folder with the first author and her supervisors having sole access.

## 3. Results

### 3.1. Overview of Themes

Five themes emerged from the analysis, namely, “*Not ready to transition*”, “*Self-management*”, “*Challenges to adherence*”, “*Psychosocial support*” and “*Adolescent-friendly services: filling the gap*” (See Table 2 below).

### 3.2. Theme 1: Not Ready to Transition

This theme depict apprehension about adult care and resistance to transition.

#### 3.2.1. Apprehension About Adult Care

Participants explained how their past experiences of the local clinic where adult HIV care is provided—which included long waiting times and often having to spend an entire day for their scheduled visits, contributing to them being hesitant to move to adult care. One adolescent reported:

*So yeah, it made me stand every month outside, cold, around 7. I’ve been standing two hours and another three hours extra inside and another four hours in the pharmacy.*

(P2, Male, 18 years)

Participants also expressed how the clinical environment evoked fears of potential exposure to illness as a result of the crowded clinic and prolonged waiting:

*I didn’t like the time management. I didn’t like sitting there. It felt like a hospital you gonna die! Yo, I didn’t like I didn’t like that fact. Like, cause every time you just got sick. And then, it’s like you go to pharmacy, eight hours sitting there, eight hours at pharmacy.*

(P6, Female, 16)

**Figure 2:**
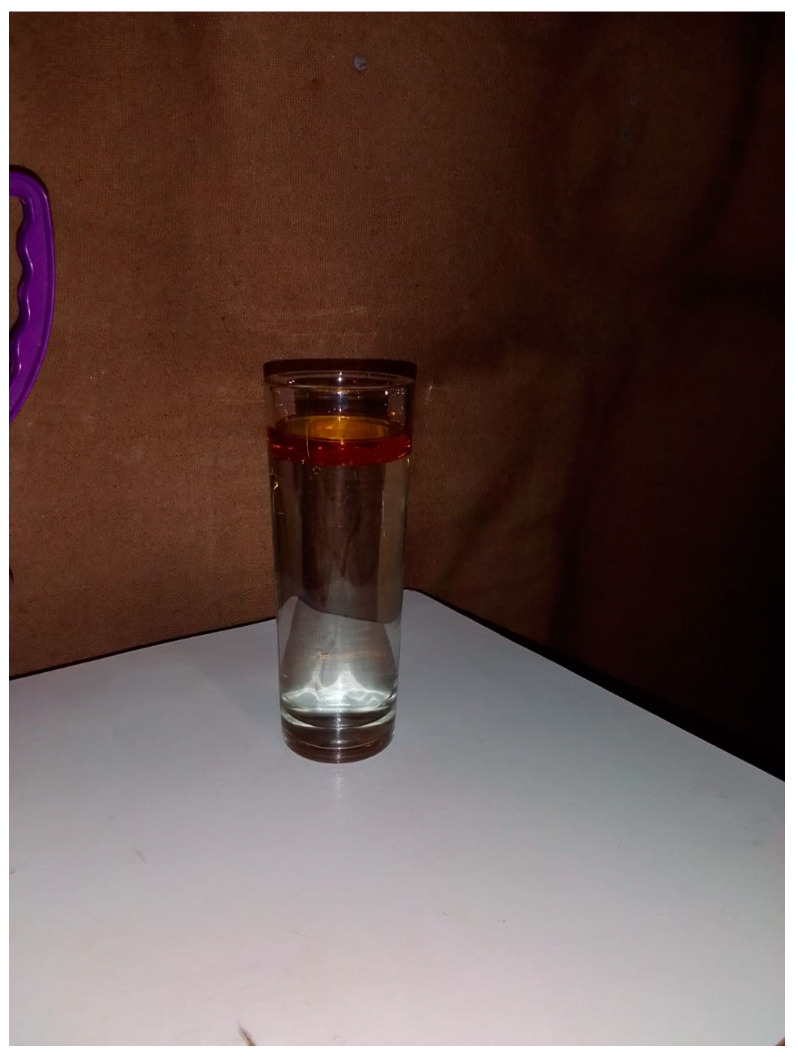
“Oil and water”.

The abovementioned sub-theme attributed a preference against the local clinic as a healthcare setting of choice. Participant 12 explained that she and the clinic are incompatible—i.e., “*do not mix*” … like oil and water, as illustrated in the picture that she took (Figure 2).

**Figure 3:**
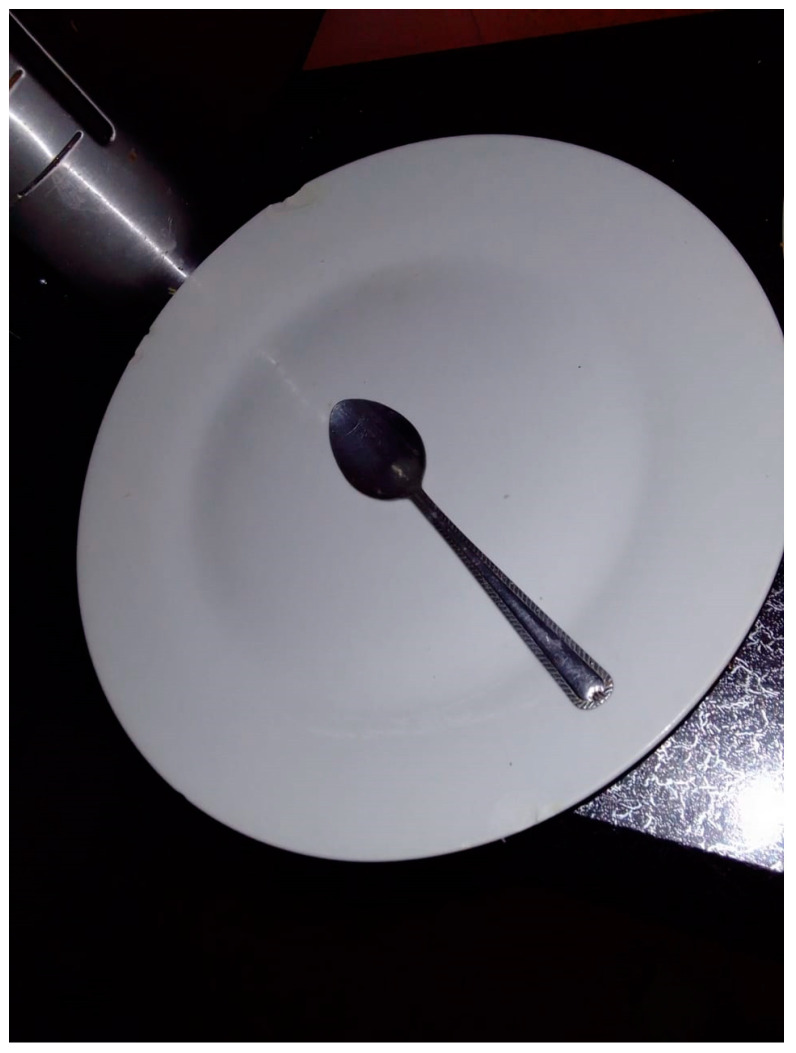
“Hungry as adults”.

Another participant mentioned that the lack of adolescent-friendly care at the local clinics leaves them physically hungry due to the prolonged waiting times. Further, she described feeling “*mentally hungry*” as the clinics failed to provide meaningful engagement, education or mental stimulation, in contrast to their current experiences at the supportive facility, referring to the picture that she took (Figure 3).

*And there you get hungry. I think in more ways than one because you won’t be getting the connection.*

(P10, Female, 19)

Adolescent participants also reported their fear of unintended disclosure of their HIV status, while “waiting” for care at the adult clinic, in the face of perpetuated stigma and discrimination in the community.

*They will split it. I don’t know how to put it, but they will split it into a thousand [different stories] so that everyone knows that you have [HIV]. That is the thing that I don’t want to talk about because people will spread it all over and it’s going to be a thing.*

(P2, Male, 18)

Moreover, participants explained that at local clinics they were seen individually by the doctor and did not interact with peers. They express this anticipated loss as a result of their previous experiences:

*I can’t be alone. I can’t return there. You see, I’m going to be alone there.*

(P16, Female, 16)

*It feels like a loss, ma’am, a big loss, ma’am, where you, sometimes when you’re alone, you think about life, you also think about, eish.*

(P21, Male, 18)

#### 3.2.2. Resistance to Transition

The participants expressed their resistance to transitioning to adult HIV services, largely due to the quality of care they currently receive. This reluctance or resistance is further reinforced through feelings of being unprepared, fear of sharing or speaking up and the anticipated loss of privacy they associate with the transition.

As discussed in the previous sub-theme, participants described the lack of provisions at the local facility, whereas currently, they explain

*Because while we’re waiting for whoever, you make your hot chocolate and eat your food. They don’t have that there.*

(P1, Female, 19)

This furthers their resistance to transfer to another facility. Another factor inhibiting willingness to transition is their difficulty speaking up:

*Yeah, it’s not easy, it’s not easy to share.*

(P4, Male, 19)

They state that the main reason for their unwillingness to transition to adult care is a certain feeling of not being “ready” to leave the facility. Participant 13 explains

*And I really love that like being feeling safe here. So, I don’t think we’re yet ready to leave here.*

(P13, Female, 14)

Participants therefore recognise that they may not feel safe at another facility, which contributes to their reluctance to leave. Additionally, participants express that they do not feel adequately prepared to transition wherein they are expected to take responsibility for their care. This is evidenced in:

*I don’t feel so exactly prepared. I like to go in situations when they come to me. I’m not a forward-thinking person, so I’m not sure about that. So, to me, that I’m not fully equipped to leave [this facility] and go to a clinic.*

(P23, Female, 18)

### 3.3. Theme 2: Self-Management

Participants displayed competency for self-management by taking responsibility for their chronic condition through acceptance of their HIV status, living healthy, management of their mental wellness, being internally motivated and sublimation and navigating personal accountability and self-reliance. This is exemplified by the quote by Participant 9, who maintained a positive self-concept in spite of perceived and/or experienced stigma:

*This picture I look at me, and I say to myself “I don’t look like the person that has HIV”. People can talk and say that I am HIV positive, but no one will see me that I am HIV positive. And I am also happy, and I am not asking myself that I should kill myself because I am HIV positive or what. It’s just that I am happy in life.*

(P9, Female, 19)

Many participants confirmed that they understood that HIV is a life-long condition and that they need to remain adherent to ART medication, as commented by Participant 2:

*I think of my medication as… my oxygen, part of my life.*

(P2, Male, 18)

Further, some participants also expressed a strong sense of independence in their development to adulthood, as illustrated by the quote below:

*Cause eventually I’m going to be an adult also… So, if I don’t like, try and depend on myself, who will [be there]? If I don’t try to motivate myself, who will motivate me? Because, like, of course, it’s my life.*

(P23, Female, 18)

Other ways that participants demonstrated self-management were through finding positive outlets to their challenges through writing, playing chess and physical exercise.

*I don’t know, um, my escape to, I don’t know, but it is like, this whole picture is an escape to the whole, to the world, because when I write, like, my diary, I just want to be alone.*

(P1, Female, 19)

Participant 21 explained that through exercise he experiences a sense of clarity and feeling “*normal*”.

### 3.4. Theme 3: Challenges to Adherence

Participating ALHIV found adherence to be challenging in various ways, with some reporting to often forget to take medication and experiencing pill fatigue.

#### 3.4.1. Physical/Tangible Challenges to Remain Adherent

**Figure 4:**
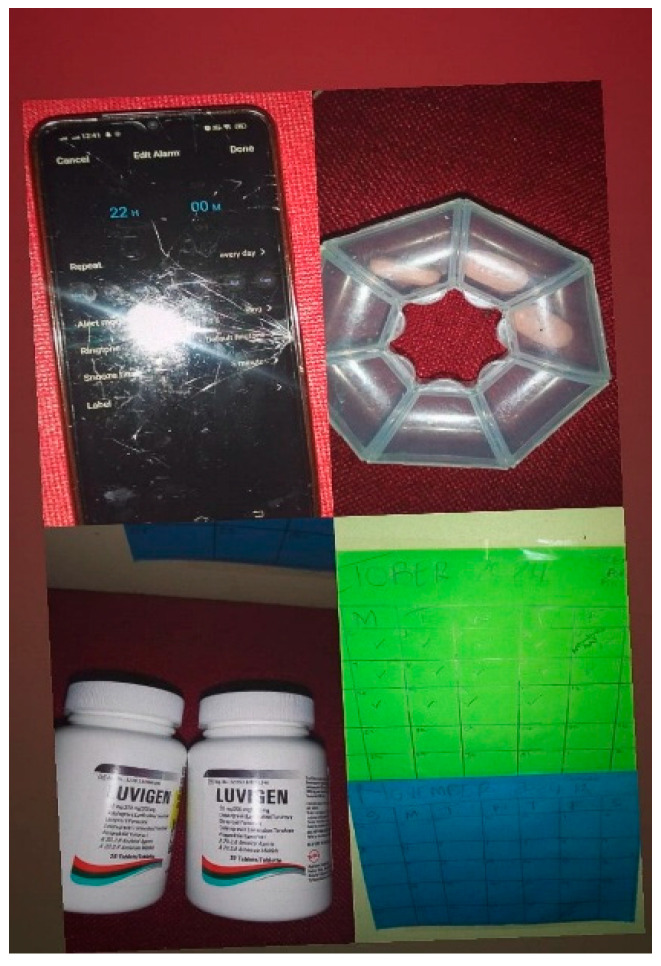
“Reminders”.

Participants attempted to mitigate the abovementioned “forgetfulness” by setting up reminders, such as alarms on their phones or calendars. As seen in the figure (Figure 4) below, one participant uses a calendar, cell phone reminder, as well as a measured pillbox to ensure that she has taken her medication.

Participants proffered that their forgetfulness may be attributed to a change in schedules over the weekends, as they usually take their medication before or after school.

*I always drink my pills from Monday to Friday. The weekend? No, I forget.*

(P15, Female, 18)

Participants admitted that they often became demotivated due to treatment fatigue, that is, having to take their ARV medication daily. Other participants reported the physical difficulties of drinking pills—such as discomfort when swallowing—increased their reluctance to take their medication as prescribed.

#### 3.4.2. Mental/Emotional Challenges to Remain Adherent

**Figure 5:**
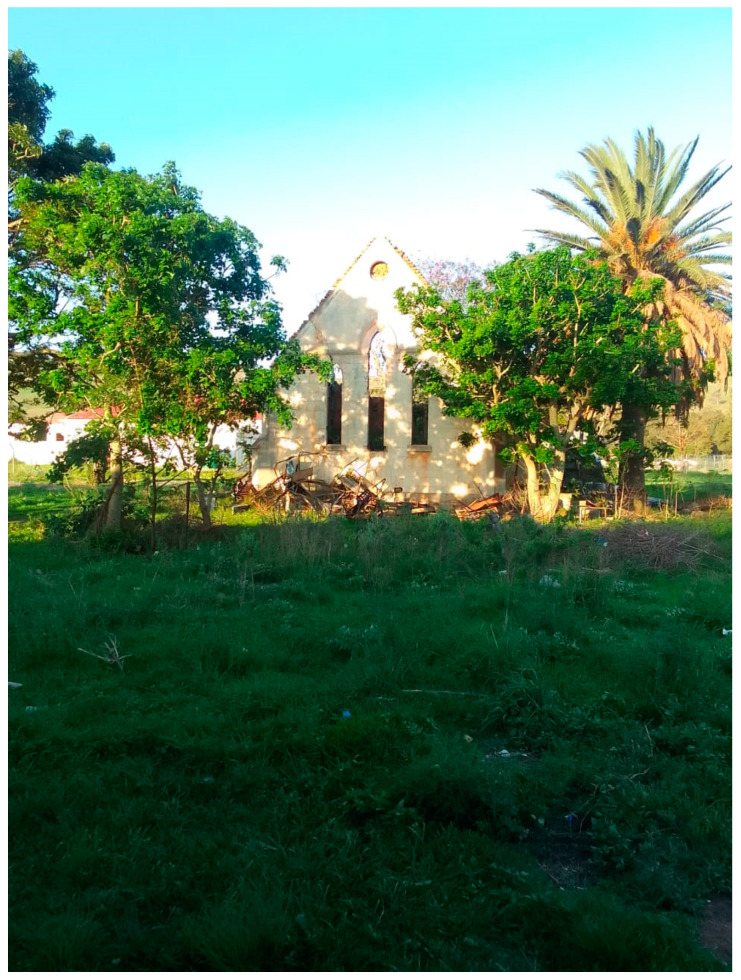
“*Yeah, it’s like literally dark because I can’t find anything that could make me happy.*” (P23, Female, 18).

Some participants described negative feelings about themselves and about HIV that negatively impacted on their motivation to remain adherent. Participant 23 illustrated her decreased self-esteem and self-worth through a picture of an abandoned building (Figure 5).

For another participant, being the only sibling who is living with HIV impacted negatively on her self-esteem.

*And the reason why I felt like I was all bad is because I have siblings. My siblings don’t have HIV. So, I was like, why me?*

(P1, Female, 19)

Another participant mentioned that living with HIV is a negative thing because of his negative experiences at the local clinic, as well as the reality of high mortality rates amongst people living with HIV.

*If it wasn’t a bad thing, why would you wake up early in the morning, stay in [the line] 4 hours, then 3 hours [in the pharmacy] extra? And walk miles to… No. It’s not a good thing. It’s a bad thing. Some people die. With just a small mistake.*

(P2, Male, 18)

In some instances, ALHIV may hold on to negative feelings about themselves and their HIV status, even when disclosed to in a sensitive manner by health workers.

### 3.5. Theme 4: Psychosocial Support

This theme discusses the various networks of support the participants have (“*positive support outside of healthcare*”) and may lack (“*insufficient support outside of healthcare*”). This includes support outside of their facility, such as from their friends and family. One participant mentioned that she does not feel supported at home because of her family’s lack of acknowledgement of her living with HIV:

*Even at home, there is no one who randomly talks about, like, HIV, like, I don’t live home like, I don’t have a family that—I can’t say acknowledge but something like they are not as supporting because my, my person who I got HIV from, he is no longer.*

(P12, Female, 19)

In contrast, some participants mentioned multiple sources of support, from family members, as well as both at home and at the facility. Participant 1 described how a family member supported her to remain adherent through a shared calendar to log her pill-taking:

*Like, see, when this month, oh, it’s close to December and I might be busy, I have to know that I give, like, my cousin my calendars. This is not my room. So, it’s my cousin’s calendar. So that when I take pills, I can tell her that, okay, I’ve taken my pills, then she ticks.*

(P1, Female, 19)

In other cases, some participants felt their caregivers were overbearing, reflecting a different end of the spectrum.

*My father was yelling at me, telling me I’m not taking the tablets. You know what happens now if you don’t take the tablets. It was the wrong medication.*

(P2, Male, 18)

*There must be a limit, yeah.*

(P3, Male, 18)

*And, yeah, I don’t like them to keep reminding me. Because I feel like I have my own strategies that I use.*

(P4, Male, 19)

As Participant 4 above explains, he prefers a limited amount of support and reminding because he attempts to rely on himself to remain adherent to his medication.

### 3.6. Theme 5: Adolescent-Friendly Services: Filling the Gap

Participants explain how the facility addresses their unique needs, particularly in fostering group connection, minimising feelings of isolation and improving psychological health. The first three subthemes in this theme explicates this through the components of the biopsychosocial framework, which posits that health can be influenced by the amalgamation of biomedical, psychological and social aspects [11,32]. The first sub-theme, “groups improve health and wellbeing through ‘the biomedical’”, discuss how knowledge about HIV and its management is shared and how adherence is encouraged.

#### 3.6.1. Groups Improve Physical Health

The peer group sessions at the facility play a significant role in fostering health and adherence to treatment amongst the participants. This is accomplished through the provision of education, support and motivation. The participants highlight the imperative role of group sessions, wherein their healthcare provider is in attendance, in equipping them with the necessary knowledge to maintain their health and understanding of HIV. This shows that participants rely on and trust their healthcare provider to supply them with factual information. To underscore the importance of a reliable source, Participant 1 explains that her trust in her healthcare provider is more beneficial than access to the Internet. Another participant emphasizes the transformative impact of such education from their healthcare provider to make informed decisions about their health:

*So, yeah, I just appreciate the education, the people, the doctors because they are the ones who put me here. There’s nothing else. Because if it wasn’t for [my doctor], I don’t know what I would have been. Maybe I would have gotten that aids or something.*

(P15, Female, 18)

In addition, healthcare providers play an important role in motivating consistent adherence. This is echoed by Participant 1 sharing her journey with adherence, which she links with her academic success:

*I didn’t expect to be where I am, first of all, and I didn’t expect that I would, I would have achieved so many, like, the, I took the picture with the tablets because it is one of the things, okay, pills are one of the things that I suffered, like, to, I was struggling very much to take the pills, so that’s why I took my certificate with the pills because of that, like, if, if, if I didn’t take the pills, I wouldn’t be here, and me taking the pills made me achieve all of those things because it was not easy, like, I mean, like, I was one of the top achievers.*

(P1, Female, 19)

Peer groups therefore significantly contribute to the health outcomes of the participants and may serve as a tool to foster adherence. This is achieved through the provision of reliable information, practical guidance from peers and promoting empowerment through shared experiences.

#### 3.6.2. Groups Improve Psychological Wellbeing

The second sub-theme illustrates how participants’ psychological health is improved through peer connection, shared strategies to remain adherent and what participants have learned to do when they are not in the facility for psychological support. Participants explain that the peer group sessions mitigated feelings of isolation and assisted in forming meaningful connections. The group ultimately created a supportive environment wherein participants felt accepted and less alone.

*There’s people for you. If your family doesn’t care about you, we care about you. You’re not alone. You’re with people and friends. You will meet friends, and you’ll meet other people also.*

(P17, Female, 18)

The facility provided the participants with psychological support through the peer group sessions; however, participants felt the necessity to develop strategies to duplicate the support they receive at this facility. One participant mentioned that she uses her diary as a way to replicate the peer support from the facility:

*So, every time I feel like, horrible, I write it in my diary, and I feel like it’s the closest thing to me to [this facility].*

(P1, Female, 19)

Other participants, similarly, attempted to reconcile the peer interaction at the clinic when they are at home through their comfort items.

#### 3.6.3. Groups Improve Social Wellbeing

The third sub-theme explains how participants’ health and wellbeing were improved through the social needs being met at the facility. Such as they have a safe space to talk about HIV, they became family with their peers in the facility, how they support one another and the sense of belonging they feel with their peer group. Further, it includes how the healthcare providers at the facility have built long-standing relationships with the participants. Participants expressed the importance of these long-standing relationships with their healthcare providers. Participant 9 explains

*So, the first time I met [my doctor], I lost hope. She held my hand, and she told me that we are with you. You’re not alone.*

(P9, Female, 19)

Therefore, this explicates the foundation of safety and reliability that is fostered at the facility, which is central to the participants’ social and emotional wellbeing. Participant 1 strengthens this, explaining how her doctor helped her in navigating a challenging time in her life. Moreover, the healthcare providers allow access to their assistance outside of the facility through social media messaging or through private, individual sessions at the facility. Participant 7 explains

*Like, I wait for the club to come here. Sometimes the sister is busy, so she can’t maybe, like, check WhatsApp, you see. So, I wait until they come. And then we go to the office, and we can talk.*

(P7, Female, 14)

**Figure 6:**
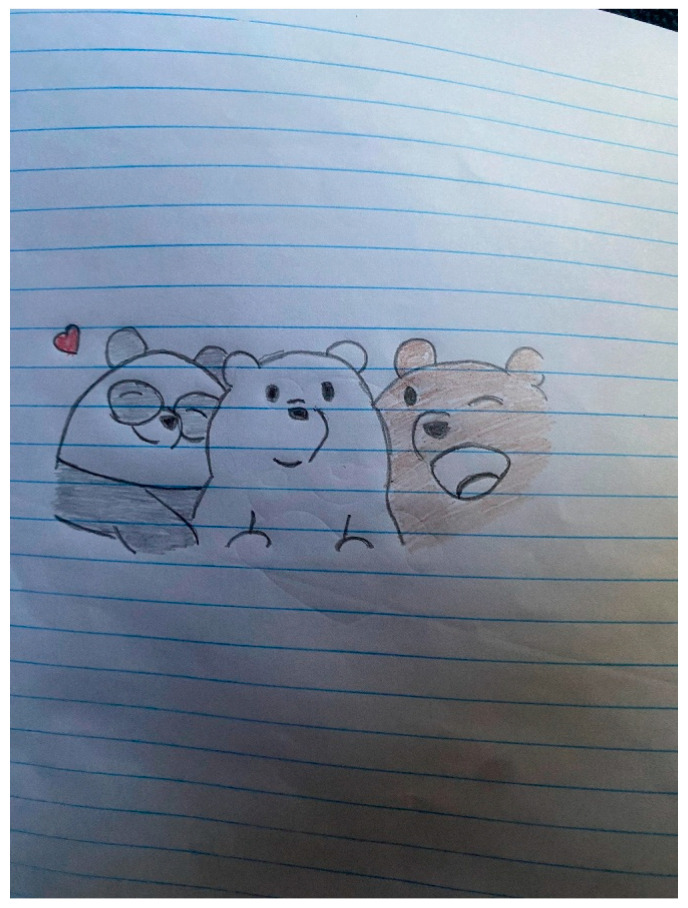
“Just another bear who found love”.

Participants further note that the groups allow for a safe space to discuss their HIV status openly. The mutual understanding of being an adolescent living with HIV empowers participants to freely share their experiences. Participants mentioned that they can only speak to their peers at the facility about HIV experiences. Alongside the mutual understanding and sense of belonging, the peer groups were often described as surrogate families, offering emotional and social support. Participant 3 furthers this by showing his picture and drawing of three bears, to illustrate the sense of family that is established between group members (see Figure 6 below).

*I ended up knowing other people, getting involved with each other, bonding, and we fell in love with each other.*

(P3, Male, 18)

Therefore, the connections formed ultimately enhance social wellbeing but also provide a support network in navigating their experiences of living with HIV.

## 4. Discussion

**Figure 7:**
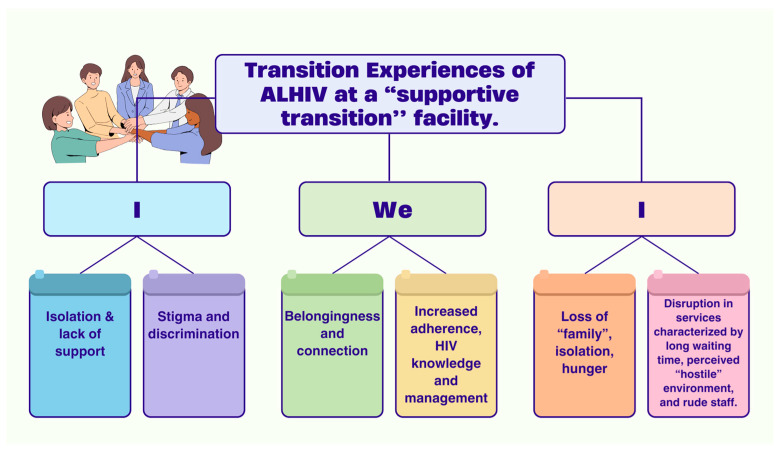
“I-We-I” Illustration.

From the qualitative findings reported above, we configured the “I-We-I” illustration to demonstrate individual ALHIV experiences *as isolated* before being transferred to the supportive facility (the first “I”); how they experience a sense of belonging and family (becoming “we”) in the supportive facility; but face apprehension about transitioning to adult care in the local clinic, where they have to be independent and self-manage (final “I”) (Figure 7).

Previous research highlights the impact of stigma, recognizing the consequences as reduced medication adherence, internalized stigma and isolation [33,34]. Feelings of isolation may increase negative feelings about living with HIV and about oneself. Evidently, negative feelings hinder engagement in care and adherence to ART [35]. Recent findings indicate the complex interplay between mental health, stigma and the lack of psychosocial support amongst ALHIV, which hinders their adherence and overall wellbeing, particularly in a post-pandemic context [6,36]. This may further exacerbate the development of mental health issues for ALHIV [6]. Moreover, long clinic visits interfere with schooling, as adolescents often have to miss a day of school for their scheduled clinic visits. This is strengthened by Zanoni et al. [37], who coined the term “Student’s Paradox” to explicate the dilemma of choosing between academics and health care. In this study, Zanoni et al. [37] find that rigid clinic schedules interrupt education for ALHIV as they need to miss classes in order to attend medical appointments. This tension not only disrupts academic progress, but may contribute to disengagement from care, particularly when adolescents prioritize their education above their health needs [37]. This is highlighting the need for more flexible, adolescent-friendly healthcare services to accommodate the dual priorities of attaining education and maintaining the HIV care cascade [37]. Previous research further strengthens the need for shorter waiting times for scheduled clinic visits at times that are more catered to adolescents’ schedules and prove more favourable to retaining ALHIV in care [4,37]. This further reinforces the need to provide services to adolescents that are accessible without infringing upon other responsibilities.

The participants in this study described the beneficial medical and psychosocial support they received at this facility. Having a scheduled clinic visit after school-going hours, peer discussion groups and a relationship with healthcare providers have proven to facilitate extended retention in care [37,38].

Participants explained that when they joined this current facility, their feelings of isolation disappeared and were replaced by feelings of belonging and connection. Peer support has proven an effective strategy to provide psychosocial support for ALHIV whilst simultaneously bridging gaps in care [39]. Moreover, it provided them with an opportunity to learn more about managing their chronic disease and how their peers manage it. Increased knowledge and deconstructing stigmatized beliefs about HIV are some of the key outcomes of the group sessions the participants took part in and played a big role in improving feelings about themselves and their chronic illness. This is strengthened by the findings of Adhiambo et al. [39], which underscores the importance of peer support as a motivating factor through the reinforcement of self-care practices and the growth of self-efficacy in managing chronic disease.

As a result of their needs being met at this facility, the question of being transferred to an adult clinic brought upon a sense of fear and apprehension, and participants reiterated their concerns that their needs might not being met in the new setting. Participants explained that they fear that they will experience loss of their support system and sense of belongingness, ultimately, returning to the initial isolation they felt in paediatric care. This highlights the current lack of services provided to ALHIV, which significantly impacts their readiness to transition to independent adult care [40]. Further, this necessitates long-term follow-up after transitioning to adult care to ensure ALHIV is retained and engaged in care [20,25]. This may be supported through models including peer support or navigation—which evidently assists patients in managing their treatment, life challenges and challenges to adherence—to maintain ALHIV in adult care [41,42,43].

## 5. Limitations of This Study

This photovoice study has some limitations, such as the sample being limited to adolescents receiving care at the selected facility, therefore cannot be applicable to settings where the same intensity of care is not provided. As a result, different experiences of transition may occur. Moreover, the selected facility is supported by external funding, whereas a large number of public health facilities are dependent on governmental funding, therefore the experiences of the study participants might not be comparable to other settings.

## 6. Conclusions

This photovoice study highlights the complex and dynamic experiences of ALHIV in their care continuum, as they move from paediatric care, adolescent-friendly services, to adult HIV services. The “I-We-I” configuration encapsulates the evolving sense of identity and support; from initial isolation to a period of belonging within a supportive environment and finally, to the apprehension of self-managing care independently at an adult facility. We underscore the critical role of adolescent-friendly, supportive services to improve adherence and psychosocial wellbeing, while also revealing persistent challenges in preparing ALHIV for adult-oriented care. Targeted interventions, such as structured preparation for the transition, peer support and mentorship and continued psychosocial support could enhance readiness to transition as well as retention in care, which will ultimately aid in improving health outcomes for ALHIV.

## Figures and Tables

**Table 1:** Description of participants.

Category	Sub-Category	Total (N = 24)
Age (in years)	13–15	8
	16–19	16
Sex	Female	15
	Male	9

**Table 2:** Thematic analysis framework.

Themes	Sub-Themes	Codes
Not ready to transition	Apprehension about adult care	Negative experiences at local clinic
		Reluctance to transition to adult care
		Anticipated loss of peer connection at local clinic
		Experiences of stigma and discrimination
		“We’ll be hungry at the adult clinic”
	Resistance to transition	Improved service at current facility
		Difficulty talking in groups
		Not ready to transition
Self-management	Taking responsibility	Acceptance of living with HIV
		Healthy living
		Management of mental wellness
		Internal motivation
		Sublimation
		Navigating personal accountability and self-reliance
		“Taking my medication is like oxygen”
Challenges to adherence	Physical/tangible challenges	Forgetting to breathe; forgetting to take medication
		Pill fatigue
	Mental/emotional challenges	Negative feelings about self
		Negative feelings about HIV
Psychosocial support	Positive support outside of healthcare	Support from family
		Multiple sources of support
	Insufficient support outside of healthcare	Overbearing caregivers
		Lack of support at home
Adolescent-friendly services: filling the gap	Groups improve physical health	Increasing HIV knowledge
		Linking adherence to personal success
		Health care provider helping adherence
	Groups improve psychological wellbeing	Group mitigating isolation
		Remembering to breathe/strategies to remain adherent
		Supplement to the facility
	Groups improve social wellbeing	Group mitigating isolation
		Remembering to breathe/strategies to remain adherent
		Supplement to the facility

## Data Availability

The raw data supporting the conclusions of this article will be made available by the authors upon request.
